# Multiple endpoint analysis of BAC-preserved and unpreserved antiallergic eye drops on a 3D-reconstituted corneal epithelial model

**Published:** 2011-03-16

**Authors:** A. Pauly, E. Brasnu, L. Riancho, F. Brignole-Baudouin, C. Baudouin

**Affiliations:** 1 INSERM, UMR_S968, Institut de la Vision, Paris, France; 2 UPMC University Paris 06, UMR_S 968, Institut de la Vision, Paris, France; 3 CNRS, UMR_7210, Paris, France; 4 Department of Ophthalmology III, Quinze-Vingts National Ophthalmology Hospital, Paris, France; 5 Department of Toxicology, Faculty of Biological and Pharmacological Sciences, Paris, France; 6 Ambroise Paré Hospital, APHP, University of Versailles Saint-Quentin-en-Yvelines, Versailles, France

## Abstract

**Purpose:**

To compare the effects of benzalkonium chloride (BAC)-preserved and unpreserved antiallergic eye drops on the human 3D-reconstituted corneal epithelial model (3D-HCE).

**Methods:**

3D-HCE were treated for 24 h followed or not by a 24 h post-incubation recovery period (24 h+24 h) with phosphate-buffered saline (PBS), 0.01% BAC, unpreserved formulations of ketotifen, N Acetyl-Aspartyl Glutamic Acid (NAAGA), cromoglycate, or BAC-preserved commercial formulations of ketotifen, olopatadine, epinastine, and levocabastine. The 3D-HCE viability was evaluated using the 3-(4,5-Dimethylthiazol-2-yl) -2,5-Diphenyltetrazolium Bromide (MTT) test at 24 h and 24 h+24 h. At 24 h, the numbers of Cluster of Differentiation 54 (CD54)- and Ki67-immunopositive cells as well as the number of apoptotic deoxynucleotidyl transferase-mediated dUTP nick-end labeling (TUNEL)-positive cells were evaluated on 3D-HCE frozen sections. The expression of the tight junction-associated protein occludin was also assessed using fluorescence confocal microscopy on flat-mounted 3D-HCE epithelia.

**Results:**

The MTT and the TUNEL tests revealed a significant decrease of cell viability and an increased apoptosis in the superficial layers of the 3D-HCE only when treated with BAC-containing formulations and in a BAC concentration-dependent manner. The expression of CD54 and Ki67 in the basal layers was also increased in this group. A concentration-dependent disorganization of occludin distribution in the epithelium treated with BAC-containing solutions was also observed. The unpreserved formulations induced effects comparable to the control.

**Conclusions:**

BAC-preserved solutions decreased cell viability and induced apoptosis in a concentration-dependent manner. Moreover, they induced CD54 expression, proliferation in the basal layers, and changes in the distribution of occludin, which is consistent with a disorganization of the tight-junctions and suggests the loss of the epithelial barrier function. On the contrary, the unpreserved solutions did not impair cell structures and viability, suggesting a better tolerance for the ocular surface. As allergic patients often exhibit impaired and inflammatory ocular surface, BAC-free compounds should be the first choice when treating allergic conjunctivitis.

## Introduction

To limit and counteract the clinical manifestations of allergic diseases, antiallergic compounds can be used. One of these molecules, ketotifen fumarate, has demonstrated both H1-receptor antagonism and mast cell stabilizing properties while inhibiting chemotaxis and eosinophil activation [1,2]. Moreover, ketotifen fumarate was shown to be well tolerated and effective in reducing the signs and symptoms of allergic conjunctivitis [3-6]. Allergic conjunctivitis, however, has often a tendency to become chronic, due to repeated allergic challenge or progressive impairment of the tear film and ocular surface [7,8].

As preservatives are usually used to prevent multidose eyedrop microbial contamination, their chronic administration may cause further ocular surface changes, at the levels of tear film and conjunctiva. They can induce cytotoxic effects and deleterious reactions when used over long-term periods. Indeed, the mostly used preservative benzalkonium chloride (BAC) was already shown to exhibit toxic and inflammatory effects in clinical, in vivo and in vitro studies [9-20]. Chronic use of BAC in eye drops is known to be responsible for apoptosis and oxidative stress on conjunctival cells, and to induce conjunctival inflammation that has demonstrated potentially harmful effects on glaucoma outcome, e.g., on glaucoma surgery efficacy [17,21-25].

In this context, the implementation of very sensitive tools to predict eye tolerance is critical for ophthalmologists, who may be faced with long-term induced toxicity of substances used at low concentration in ophthalmic preparations. Supplied by SkinEthic^®^ Laboratories (Nice, France), the reconstructed three-dimensional (3D) model of human corneal cells (3D-HCE) is an appropriate model for pre-screening or investigating the undesirable effects of ophthalmic drugs. It constitutes an interesting alternative to animal testing that is time-consuming and often invasive and may lack suited sensitive tools able to detect subclinical reactions [26-28]. Multi-endpoint analyses using adapted and improved techniques on such 3D-models have already proved efficacy for the assessment of BAC toxicity [28] and eyedrop tolerance [27].

The objective of this study was to investigate a large range of commonly used antiallergic eye drops in this 3D-HCE system and compare the tissue changes after treatment with BAC-preserved commercial formulations of ketotifen, olopatadine, epinastine or levocabastine, and unpreserved commercial formulations of ketotifen, N Acetyl-Aspartyl Glutamic Acid (NAAGA), or cromoglycate. Particularly, our purpose was to determine the involvement of BAC in epithelial cell damage induced after treatment with BAC-preserved and unpreserved antiallergic eyedrops.

## Methods

### Tissue model and antiallergic solution treatments

The 3D-HCE model (SkinEthic^®^ Laboratories, Nice, France) consists of immortalized HCE cells grown vertically on a 0.5 cm^2^ insert permeable polycarbonate filter. All the experiments were conducted as published previously [27-29]. Thirty microliters of each solution were applied on the apical surface of 3D-HCEs for 24 h and 24 h followed by 24 h additional recovery time: sterile phosphate-buffered saline (PBS) used as negative control solution, BAC solutions at 0.01% used as positive control, the commercial solutions of 0.01% BAC-containing ketotifen fumarate 0.025% (Zaditen^®^; Novartis Pharma SAS, Rueil-Malmaison, France), 0.01% BAC-containing olopatadine chlorhydrate 0.1% (Opatanol^®^; Patanol^®^; Alcon, Ft. Worth, TX), 0.01% BAC-containing epinastine chlorhydrate 0.05% (Purivist^®^; Allergan, Irvine, CA), 0.015% BAC-containing levocabastine chlorhydrate 0.05% (Levophta^®^; Chauvin Bausch & Lomb, Montpellier, France), preservative-free ketotifen fumarate 0.025% (Zalerg^®^; Thea, Clermont-Ferrand, France), preservative-free NAAGA 4.9% (NAABAK^®^; Thea) and preservative-free sodium cromoglycate 2% (Cromabak^®^; Thea; Table 1).

**Table 1:** Benzalkonium chloride (BAC) and active compound content of the antiallergic eye drops tested.

**Eye drops**	**Active compound content**	**BAC content**
Ketotifen fumarate (Zaditen®; Novartis Pharma SAS,Rueil-Malmaison, France)	0.025%	0.01%
Olopatadine chlorhydrate (Opatanol®; Patanol®; Alcon, Ft. Worth, TX)	0.1%	0.01%
Epinastine chlorhydrate (Purivist®; Allergan, Irvin, CA)	0.05%	0.01%
Levocabastine chlorhydrate (Levophta®; Chauvin Bausch & Lomb, Montpellier, France)	0.05%	0.015%
Preservative-free ketotifen fumarate (Zalerg®; Thea, Clermont-Ferrand, France)	0.025%	-
Preservative-free NAAGA (NAABAK®; Thea, Clermont-Ferrand, France)	4.9%	-
Preservative-free sodium cromoglycate (Cromabak®; Thea, Clermont-Ferrand, France)	2%	-

The recovery period (24 h) was chosen to assess the potential reversibility of toxic effects on 3D-HCE. Six series of 3D-HCE were used for each solution: two series for cell viability 3-(4,5-Dimethylthiazol-2-yl) -2,5-Diphenyltetrazolium Bromide (MTT) testing, two series for histomorphologic analyses after hematoxylin and eosin staining and immunohistological analyses on cryosections, and two series for immunofluorescent labeling on the most superficial layers of 3D-HCE by en-face confocal microscopic analyses.

### Modified MTT test

The modified MTT test was used to assess cellular viability as described previously [27-29]. Experiments were conducted in duplicate. The 3D-HCEs were transferred in 24-well plates containing 300 μl of the MTT solution diluted at 0.5 mg/ml in culture medium and 300 µl of the same MTT solution were applied on the apical surface of the 3D-HCEs. Reconstituted tissues were incubated for 3 h. Then, the 3D-HCEs were transferred into 24-well plates containing 750 µl isopropanol, and 750 µl isopropanol were added to the apical surface of the 3D-HCEs. After a 2-h agitation, solutions were vigorously homogenized before reading the absorbance at 570 nm versus 690 nm. Results were expressed as a percentage of cell viability compared to the negative control, PBS. Analyses were performed using Safire technology (Tecan, Lyon, France).

### Confocal immunofluorescence analyses on cryosections and entire epithelia

After incubation with the 9 different solutions, the 3D-HCE samples were transferred into Petri dishes containing PBS to be separated into two pieces using a surgical scalpel. Each piece of tissue was embedded in OCT^®^ medium (Tissue-Tek, Miles Inc., Elkhart, IN), and frozen at –80 °C. Vertical cryosections (10 μm thick) were then cut using a cryotome (Leica CM 3050s, Leica Microsystems AG, Wetzlar, Germany). The cryosections were fixed in 4% paraformaldehyde (PFA) for 20 min before immunofluorescent labeling of the tight junction protein occludin.

### Detection of apoptosis (TUNEL assay), inflammation (CD54) and proliferation (Ki67) on 3D-HCE cryosections

#### Apoptosis, TUNEL assay

Apoptosis in the tissue layers was detected using a terminal deoxynucleotidyl transferase-mediated dUTP-nick end labeling (TUNEL) kit containing TUNEL enzyme and TUNEL label (Roche Diagnostics, Meylan, France). Nuclei were stained with 4',6-diamidino-2-phenylindole (DAPI) and the cryosections were mounted in an anti-fade medium (Vectashield; Vector Laboratories, Burlingame, CA).

#### CD54 (ICAM-1) and Ki67 immunostaining

First, samples were fixed with 4% PFA for 10 min. Then, samples were permeabilized with 0.01%-diluted Triton X100^®^ (Sigma Chemical Company, St. Louis, MO) for 5 min. Cells were incubated in presence of the mouse anti-human cluster of differentiation 54 (CD54) (IgG1; 1:100 final dilution; BD Biosciences, PharMingen, San Diego, CA), the mouse anti-human Ki67 (1:25 final dilution; Immunotech, Marseilles, France) or with the isotypic control mouse IgG1 (BD Biosciences) primary antibodies. Alexa 488 conjugated-goat anti-mouse IgG (Invitrogen-Molecular Probes, Eugene, OR) was used as second antibody at a 1:500 dilution. Nuclei were labeled with propidium iodide (PI) and cryosections were mounted in Vectashield. Samples were analyzed under a laser confocal microscope equipped with a digital camera (E800; PCM 2000; Nikon, Champigny-sur-Marne, France). Immunopositive cells were then counted under the 20× objective of the microscope in three different areas. Results were calculated as the average of counts, and finally expressed as cell numbers per mm of epithelial length (mm.E.L.) after each treatment.

#### Confocal immunofluorescence on entire epithelia for tight junction staining

The rabbit anti-human occludin (IgG1; 1:100 dilution; Dako, Glostrup, Denmark) was used for tight junction staining. Alexa 488-conjugated goat anti-rabbit was used as second antibody. Samples were then analyzed under a laser confocal microscope (E800; PCM 2000; Nikon) for detecting occludin expression.

### Quantification and statistical analysis

Quantification of TUNEL-, ICAM-1-, and Ki67-positive cells was performed manually, using a microscopic grid on images under 400× magnification. Results were expressed as mean cell numbers per millimeter of epithelial length (mm.E.L). Standard deviations were indicated. Statistical comparisons were performed using two-way analysis of variance (ANOVA), followed by multiple pairwise comparisons using the Fisher’s adjustment (Statview V for Windows; SAS Institute, Cary, NC).

## Results

### Cell viability: MTT test

**Figure 1:**
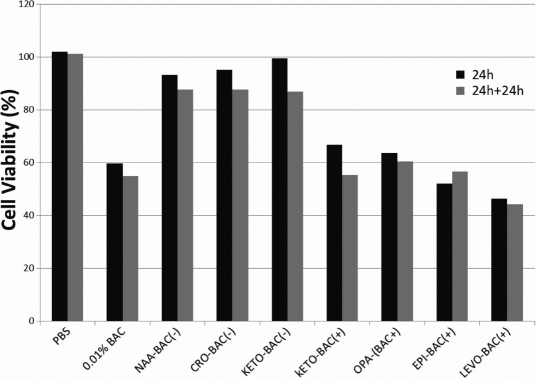
Cell viability MTT test. Cellular viability of 3D-HCEs treated with PBS, 0.01% BAC, NAA-BAC(-), CRO-BAC(-), KETO-BAC(-), KETO-BAC(+), OPA-BAC(+), EPI-BAC(+), or LEVO-BAC(+) for 24 h followed or not by a 24 h post-incubation period (24 h+24 h-recovery). BAC induced a concentration-dependent decrease of cellular viability. At 24 h, the unpreserved NAA-BAC(-), CRO-BAC(-), and KETO-BAC(-) formulations induced a slight or insignificant decrease of cellular viability, while the KETO-BAC (+), OPA-BAC(+), EPI-BAC(+), and LEVO-BAC(+) BAC-containing formulations induced a marked decrease of cellular viability compared to control. After the 24-h recovery period, the unpreserved formulations showed a weak additional decrease of cellular viability, while the BAC-containing formulations still induced a strong decrease of cellular viability compared to control, showing irreversible damage to 3D-HCE. Results are expressed as percentage of cell viability compared to the PBS control.

The PBS negative control did not affect the cell viability neither at 24 h nor after the 24 h-recovery period (24 h+24 h; Figure 1). The unpreserved formulation of ketotifen fumarate KETO-BAC(-) showed the same level of cell viability as PBS at 24 h (99,4%) and a slight decrease of viability after 24 h+24 h (86.9% of the control). The preservative-free formulations of NAAGA and cromoglycate, NAA-BAC(-) and CRO-BAC(-), also showed a weak decrease of cellular viability at 24 h (93.2% and 95.1%, respectively) and after 24 h+24 h (87.7% for both; Figure 1).

Conversely, as expected according to previous studies [28], 0.01% BAC showed a significant decrease of cell viability at 24 h and after 24 h+24 h (59.6% and 55% viability, respectively). Cell viability decreased in a BAC-concentration dependant manner for the BAC-containing antiallergic formulations, with a highest toxicity observed with the 0.015% BAC-containing levocabastine chlorhydrate 0.05% [LEVO-BAC(+)]. Cell viability levels were 66.8% at 24 h and 55.3% at 24 h+24 h for 0.01% BAC-containing ketotifen fumarate 0.025% [KETO-BAC(+)], 63.7% at 24 h and 60.5% at 24 h+24 h for 0.01% BAC-containing olopatadine chlorhydrate 0.1% [OPA-BAC(+)], 52.1% at 24 h and 56.6% at 24 h+24 h for 0.01% BAC-containing epinastine chlorhydrate 0.05% [EPI-BAC(+)], and 46.3% at 24 h and 44.3% at 24 h+24 h for LEVO-BAC(+).

### Immunofluorescence analyses and quantification of apoptosis (TUNEL)

**Figure 2:**
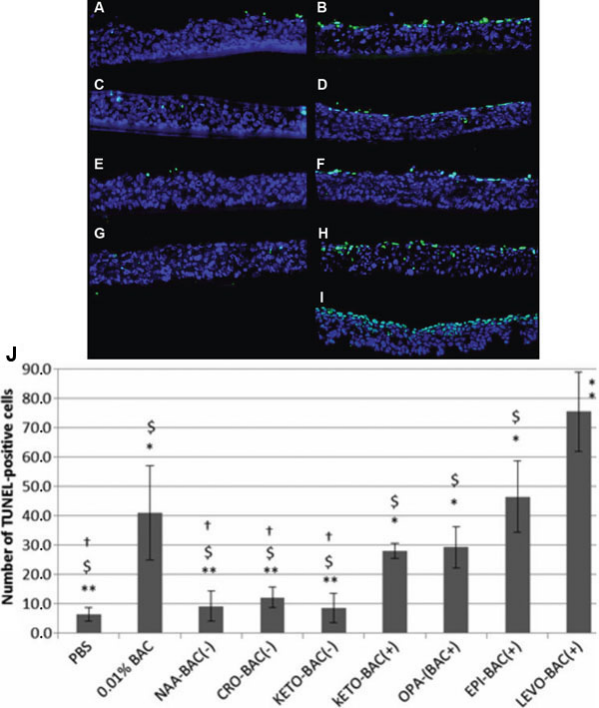
Apoptosis analysis (TUNEL). Localization of TUNEL positive cells (green) on 3D-HCE samples after 24 h of incubation with PBS (**A**), 0.01% BAC (**B**), KETO-BAC(-) (**C**), KETO-BAC(+) (**D**), NAA-BAC(-) (**E**), OPA-BAC(+) (**F**), CRO-BAC(-) (**G**), EPI-BAC(+) (**H**), LEVO-BAC(+) (**I**). Nuclei were stained with DAPI (blue). No or very rare apoptotic cells were observed after PBS (**A**), KETO-BAC(-) (**C**), KETO-BAC(+) (**D**) and NAA-BAC(-) (**E**) treatments. KETO-BAC(+) (**D**) and OPA-BAC(+) (**F**) induced moderate expression of apoptosis, and 0.01% BAC (**B**), EPI-BAC(+) (**H**) and LEVO-BAC(+) (**I**) induced a greater number of TUNEL-positive cells principally in the apical cell layers, and also in the middle epithelial layers with EPI-BAC(+) (**H**) and LEVO-BAC(+) (**I**). Deeper modifications were observed with 0.015% BAC-containing LEVO-BAC(+) (**I**) compared to 0.01% BAC (**B**), with a greater number of TUNEL-positive cells in the middle epithelial layers and a higher level of vacuolization in the basal epithelial layers observed with LEVO-BAC(+) (**I**). The quantification of apoptotic cells with the TUNEL assay (**J**) showed that apoptotic cell number increased in a BAC concentration-dependent manner. BAC at 0.01% and the four BAC-containing formulations KETO-BAC(+), OPA-BAC(+), EPI-BAC(+) and LEVO-BAC(+) showed much higher expression of apoptotic TUNEL-positive cells than did the unpreserved formulations NAA-BAC(-), CRO-BAC(-), KETO-BAC(-) at 24 h. Results are expressed as cell number per mm of epithelial length (mm.E.L.): Mean±SD *Statistically significant compared to PBS with p<0.0014. **Statistically significant compared to 0.01% BAC with p<0.0014. †Statistically significant compared to EPI-BAC(+) with p<0.0014. $Statistically significant compared to LEVO-BAC(+) with p<0.0014.

Few apoptotic cells were observed after PBS incubation (6.4 cells/mm.E.L.). Similar levels of apoptosis were observed with the 3 unpreserved antiallergic formulations (Figure 2): 9.2 cells/mm.E.L. for NAA-BAC(-), 12.2 cells/mm.E.L. for CRO-BAC(-), and 8.6 cells/mm.E.L. for KETO-BAC(-), without any statistically significant difference compared to PBS.

Consistent with previously published reports with the same technique [28,29], BAC at 0.01% significantly increased the number of TUNEL-positive cells compared to PBS (p<0.0014). Apoptosis also increased on cells treated with all BAC-containing antiallergic formulations, with a statistically significant difference compared to PBS (p<0.0014): 28, 29.3, 46.6, and 75.5 cells/mm.E.L. for KETO-BAC(+), OPA-BAC(+), EPI-BAC(+), and LEVO-BAC(+), respectively.

### Immunofluorescence analyses and quantification of the inflammation marker ICAM-1 (CD54)

**Figure 3:**
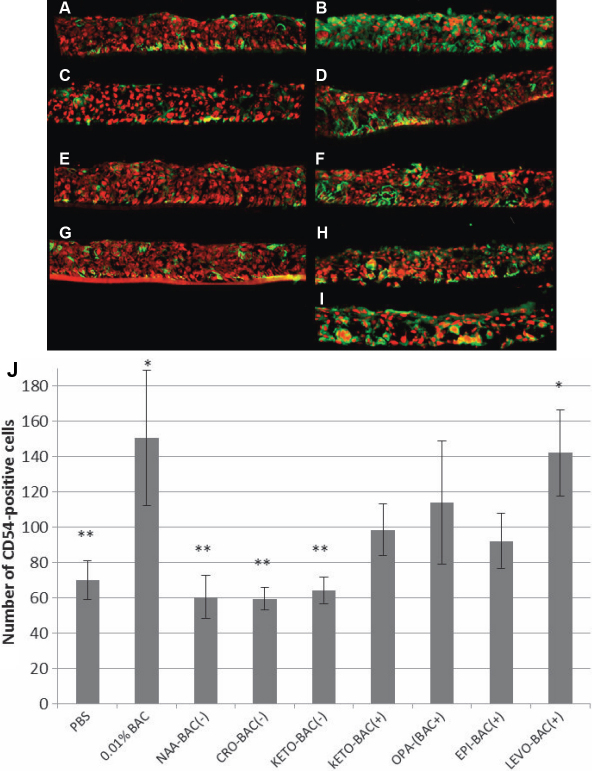
Inflammation analysis. Immunolocalization of CD54 (ICAM-1) positive cells (green) on 3D-HCE samples after 24 h of incubation with PBS (**A**), 0.01% BAC (**B**), KETO-BAC(-) (**C**), KETO-BAC(+) (**D**), NAA-BAC(-) (**E**), OPA-BAC(+) (**F**), CRO-BAC(-) (**G**), EPI-BAC(+) (**H**), LEVO-BAC(+) (**I**). Nuclei were stained with propidium iodide (PI, red). PBS (**A**), KETO-BAC(-) (**C**), NAA-BAC(-) (**E**) and CRO-BAC(-) (**G**) showed a weak expression of CD54. A significant increase of CD54 expression was observed after the treatments with 0.01% BAC (**B**) and LEVO-BAC(+) (**I**), showing a green staining in all the epithelial layers. LEVO-BAC(+) (**I**) showed deeper modifications with a higher loss of continuity between cells and a higher level of vacuolization observed in the basal epithelial layers. KETO-BAC(+) (**D**), OPA-BAC(+) (**F**) and EPI-BAC(+) (**H**) showed an intermediate CD54 expression that was localized in all epithelial layers. Quantification of CD54-positive cells (**J**) showed a higher CD54 expression with BAC at 0.01% or the four BAC-containing formulations KETO-BAC(+), OPA-BAC(+), EPI-BAC(+) and LEVO-BAC(+) than with the unpreserved formulations NAA-BAC(-), CRO-BAC(-), KETO-BAC(-) at 24 h. *Statistically significant compared to PBS with p<0.001. **Statistically significant compared to 0.01% BAC with p<0.001. Results are expressed as cell number per mm of epithelial length (mm.E.L.): Mean±SD.

CD54 expression was measured at 70 cells/mm.E.L. on PBS-treated 3D-HCE cultures (Figure 3). The three unpreserved antiallergic formulations NAA-BAC(-), CRO-BAC(-), and KETO-BAC(-) expressed CD54 at low levels too, respectively, 60.3, 59.3, and 64 cells/mm.E.L., with no statistically significant differences compared to PBS. Conversely, 0.01% BAC and LEVO-BAC(+) showed increased levels of CD54 expression with a statistically significant difference compared to PBS (p<0.001): 150.4 cells/mm.E.L. for 0.01% BAC and 142 cells/mm.E.L. for LEVO-BAC(+). KETO-BAC(+), OPA-BAC(+), EPI-BAC(+) showed increased levels of CD54 expression too, but no statistically significant difference was found neither with PBS nor with 0.01% BAC i.e., 98.5, 114 and 92 cells/mm.E.L. for KETO-BAC(+), OPA-BAC(+) and EPI-BAC(+), respectively.

### Immunofluorescence analyses of cell proliferation marker Ki67

**Figure 4:**
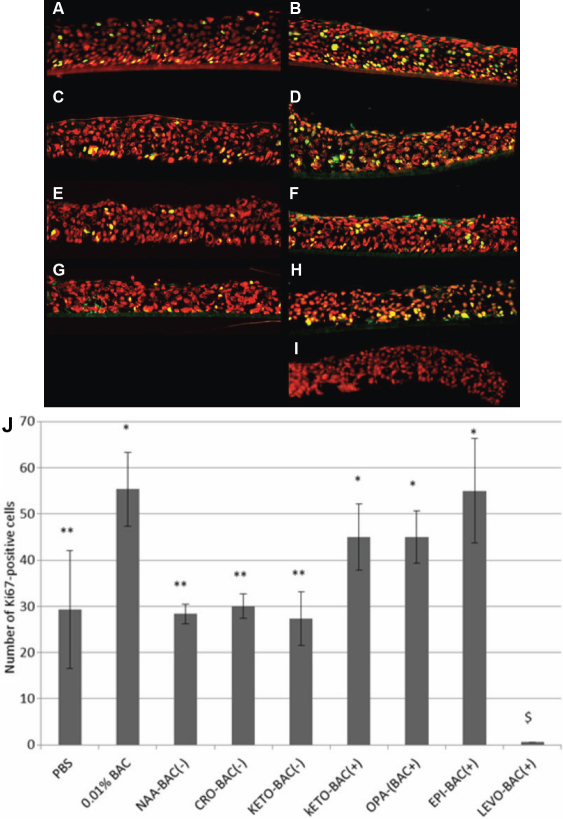
Proliferation analysis. Immunolocalization of Ki67 positive cells (green) on 3D-HCE samples after 24h of incubation with PBS (**A**), 0.01% BAC (**B**), KETO-BAC(-) (**C**), KETO-BAC(+) (**D**), NAA-BAC(-) (**E**), OPA-BAC(+) (**F**), CRO-BAC(-) (**G**), EPI-BAC(+) (**H**), LEVO-BAC(+) (**I**). Nuclei were stained with propidium iodide (PI, red). PBS (**A**), KETO-BAC(-) (**C**), NAA-BAC(-) (**E**) and CRO-BAC(-) (**G**) showed a weak expression of Ki67 in all epithelial layers. BAC at 0.01% (**B**), KETO-BAC(+) (**D**), OPA-BAC(+) (**F**), and EPI-BAC(+) (**H**) showed a higher Ki67 expression in all epithelial layers too. With LEVO-BAC(+), no Ki67 positive cells were observed, most likely due to the deep impairment of corneal cells with a most likely inhibition of proliferative capabilities of 3D-HCE submitted at this higher concentration in BAC. Quantification of Ki67-positive cells was concordant with these observations (**J**). *Statistically significant compared to PBS with p<0.04. **Statistically significant compared to 0.01% BAC with p<0.001. $Statistically significant compared to the other solutions tested. Results are expressed as cell number per mm of epithelial length (mm.E.L.): Mean±SD.

After PBS treatment (Figure 4), few proliferating cells were observed (29.2 cells/mm.E.L), scattered throughout the entire epithelium. Similar findings were observed with the unpreserved antiallergic treatments, with no statistically significant differences compared to PBS: 28.3, 30.0, and 27.3 cells/mm.E.L. for NAA-BAC(-), CRO-BAC(-), and KETO-BAC(-), respectively (Figure 4). Conversely, numerous proliferating cells, with a greater number located in the basal layer, were found after 0.01% BAC, KETO-BAC(+), OPA-BAC(+), and EPI-BAC(+) with a statistically significant difference (p<0.04) compared to PBS: 55.3, 45, 45, and 55 cells/mm.E.L., respectively. With LEVO-BAC(+), no proliferative cells were observed, most likely due to the deep impairment of corneal cells as this group showed the most important number of apoptotic cells.

### En-face confocal microscopic analysis of the tight junction-associated protein occludin

**Figure 5:**
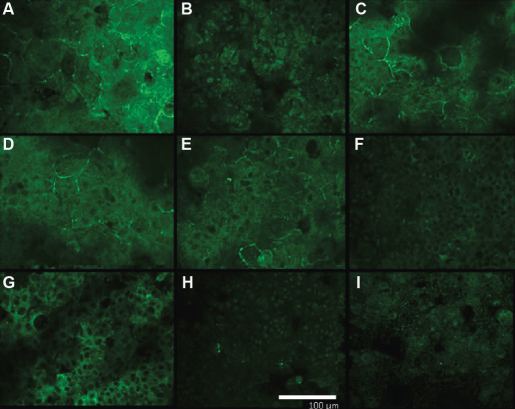
Tight junction-associated protein occludin. Immunofluorescence analysis of occludin expressions using en-face confocal microscopy after treatment with PBS (**A**), 0.01% BAC (**B**), NAA-BAC(-) (**C**) CRO-BAC(-) (**D**), KETO-BAC(-) (**E**), KETO-BAC(+) (**F**), OPA-BAC(+) (**G**), EPI-BAC(+) (**H**), LEVO-BAC(+) (**I**). Bar=100 µm.

En-face confocal microscopic analysis of 3D-HCE cultures treated with PBS, NAA-BAC(-), CRO-BAC(-), and KETO-BAC(-) revealed a fine membrane immunostaining of occludin in large superficial cells, forming a ring around the cells (Figure 5). This kind of occludin expression clearly disappeared after treatment with either 0.01% BAC, KETO-BAC(+), OPA-BAC(+), EPI-BAC(+), or LEVO-BAC(+), all showing damaged cells with non-specific staining.

## Discussion

In this study, the toxicological model of 3D-reconstructed cornea was very helpful to demonstrate the effects of BAC-preserved solutions on corneal cells in vitro, showing increased apoptosis, CD54 expression, proliferation in the basal layers and changes in the distribution of occludin induced with BAC-containing antiallergic treatments. On the contrary, the unpreserved ketotifen, NAAGA and cromoglycate solutions did not impair cell structures and viability, suggesting a better tolerance for the ocular surface.

The highly differentiated, three-dimensional epithelial system of human ocular origin is a desirable model for pre-screening or investigating the effects of ophthalmic drugs. It frees the experimenter from interspecies differences and allows a better approach to the ocular epithelial physiology than monolayer models and cells originating from other organs. It also constitutes an interesting alternative to animal testing, respecting the ethical guidelines of animal experimentation, especially the 3R rule (refining, reducing and replacing the use of animals) [30,31]. The reconstructed three dimensional (3D) model of human corneal cells (3D-HCE), supplied by SkinEthic^®^ Laboratories, was found to resemble the corneal epithelium of the human eye in morphology and thickness [32]. Such a 3D-system models is not only useful to demonstrate the different effects of toxic substances on specific cell types, but also shows the interactions between the cells and the spatial effects induced by the toxic. Moreover, epithelium cultures at the air-liquid interface are easy-to-handle and facilitate in vivo-like product exposures. The 3D-HCEs were found to express cytokeratin-3 and include hemidesmosomes within the basal layers. Furthermore, they can inhibit the flow of ionic material such as Na-fluorescein across their surface [32,33], suggesting the presence of a functional epithelial barrier. Different types of intercellular junctions have been identified in the corneal epithelium ex vivo. Among them, adherens junctions, comprising the E-cadherin protein, serve to anchor cells together [34]. Also, the tight-junctions, originally defined as zonula occludentes (ZO) and comprising occludin, ZO-1 and other proteins, are thought to provide the hydrophobic barrier preventing the free passage of molecules between adjacent epithelial cells [35-37]. In a previous study [28], we developed a new procedure of the classical MTT test used on 3D-reconstituted epidermal and corneal models to evaluate the viability. This procedure showed increased sensitivity levels and allowed detecting slight damage even in the most superficial layers. Therefore, it is well suited to the prediction of low to very low irritant potential, especially when products are used repeatedly during long-term periods of time, like in allergic conjunctivitis, when repeated allergenic challenge or ocular surface impairment occur and require sustained therapy.

Although the morphological relevance and sensitivity of the 3D-HCE model allowed the modeling of cumulative effects that may approach conditions obtained after long-term application of eye-drops [27], our in vitro findings cannot fully be extrapolated to in vivo conditions. Indeed, preserved eye drops may be less toxic in vivo, according to the continuous action of the eyelids, the permanent renewal of ocular surface epithelia, and the presence of the preocular mucin layer and glycocalyx. Conversely, the accumulation of BAC-containing eye drops in the eye and the long-term use of eye drops in allergic patients with ocular surface disorders will emphasize the risk of toxic reactions and further contribute to inflammatory stimulation throughout the ocular surface, at least at a subclinical level [38].

In the present study, using our modified MTT procedure, we evaluated the effects of either preserved or unpreserved antiallergic formulations on cellular viability and correlated these results with those of a TUNEL assay performed on 3D-HCE frozen sections. Then, we investigated on entire 3D-HCE and using en-face confocal microscopy the changes of expression and spatial distribution of cellular markers involved in intercellular junctions such as occludin after the different antiallergic treatments.

Thus, with this procedure, we were able to demonstrate concentration-dependent cytotoxic effects of BAC at 24 h, the absence of significant cellular viability decrease following treatment with the ketotifen, NAAGA and cromoglycate BAC-free formulations, and a cell viability decrease similar to that disclosed by the 0.01% BAC treatment with the BAC-containing antiallergic formulations of ketotifen, olopatadine, epinastine and levocabastatine. We confirmed the toxic and proinflammatory effects of the BAC-containing solutions using a TUNEL assay and CD54 immunostaining performed on 3D-HCE frozen sections and found a significantly increased number of apoptotic cells and an increased CD54 expression following exposure to 0.01% BAC and BAC-containing solutions compared to the control. Finally, we examined the integrity of the structural and functional barrier conferred by the tight-junctions by assessing the distribution pattern of the occludin protein. The tight-junctions regulate the passive movement of fluids, electrolytes, macromolecules and cells through the paracellular pathway, thereby contributing to the corneal defense system and to the maintenance of the corneal homeostasis. In the mouse cornea, the occludin distribution pattern was already described as altered by a detergent treatment (Triton X100) using immunohistochemistry [39]. In a previous study, Chuan et al. [40] showed the effects of contact lens multipurpose solutions on the corneal cells’ barrier function using fluorescein permeability assay and immunofluorescent staining for tight junctions proteins (ZO-1 and occludin). Recently, we also demonstrated that occludin mRNA expression was correlated to BAC early toxic effects [28]. Our results were consistent with those studies, showing the disturbance of occludin tight-junction protein distribution after BAC-containing antiallergic treatments.

Currently, allergic conjunctivitis incidence is increasing in developed countries. According to Manners T et al. [41], 15% of eye related consultations in general practice are due to allergic conjunctivitis. Recommended topical treatments for symptoms of allergic conjunctivitis include topical mast cell stabilizers and/or topical antihistamines (H1-receptor antagonists). Some of the new antiallergic drugs now available may have both effects and sometimes additional properties, such as the ability to inhibit the expression of cell adhesion molecules (CAMs) on the cell surface or to attenuate inflammatory mediator release [1,3-6,42-45].

The panel of eye drops tested in the present study was deemed to be fairly representative of the predominantly prescribed therapeutic antiallergic molecules at the time of these experiments in France. Indeed, among the commercial antiallergic eye drops, one can distinguish between two types of formulations, according to the presence of BAC as preservative. Currently, the antihistamines olopatadine, epinastine and levocabastine are available only as preserved solutions whereas ketotifen is recently accessible in both formulations. These four antihistamines constitute a group of comparable products from the therapeutic use viewpoint and all of them are available as preserved solutions. We deemed it interesting to add the preservative-free ketotifen solution in the comparison. Naaga and cromoglycate eye drops belong to a different class of antiallergic agents, i.e., the mast cells degranulation inhibitors. Although they were both available as preserved and unpreserved formulations, we chose to only test their unpreserved formulations in order not to weigh the experiment down all the more since the preserved eye drop forms of these two molecules are now much less used in therapeutics than their unpreserved counterparts. Overall, our panel choice was conducted by the actuality of the antiallergic armamentarium that is available to the patients.

There is currently enough evidence from clinical, in vivo, and in vitro studies that long-term use of preserved topical drugs may induce several deleterious effects on ocular surface, being responsible for ocular discomfort, tear film instability, conjunctival inflammation, subconjunctival fibrosis and epithelial apoptosis [9]. Several studies have confirmed the participation of high concentrations of BAC-preserved eye drops in induction of ocular surface inflammation, allergy, fibrosis, punctate corneal staining, and dry eye syndrome [9,38,46,47]. Three mechanisms have been described: detergent effects inducing loss of tear film stability; immunoallergic reactions; and direct toxic effects to epithelial cells [48,49]. Other experimental and clinical studies have shown that the long-term use of BAC-containing ophthalmic solutions can induce conjunctival stroma infiltrates and overexpression of inflammation- or apoptosis-related molecules, such as class II antigen HLADR, ICAM-1, Fas antigen, or the apoptotic marker Apo 2.7 [50-52].

In the present study, we showed that BAC-containing eye antiallergic solutions may decrease cell viability, induce apoptosis, ICAM-1 expression and proliferation in the basal layers, and changes in the distribution of occludin. Conversely, the unpreserved ketotifen, NAAGA and cromoglycate formulations did not impair cell structures and viability, suggesting a better tolerance for the ocular surface. These findings were consistent with several previous in vitro or ex vivo studies that demonstrated BAC toxicity and potential advantages of BAC-free formulations [9]. Moreover, the present results support our earlier findings on antiallergic preserved and unpreserved eye-drops. Indeed, in a previous study, we showed that antiallergic eye drops preserved with BAC induced high ICAM-1 expression levels, apoptosis and oxidative stress and reduced cellular viability in opposition to the unpreserved formulations of NAAGA and cromoglycate [53].

In addition, our results were consistent with a recent study by Ayaki et al. [54] on corneal and conjunctival cell lines that showed that cell toxicity was mostly affected by the concentration of BAC rather than the active component of antiallergic ophthalmic solutions. The use of a large range of commonly antiallergic eye drops in the present study was useful to support this point, showing BAC-concentration dependent toxic effects in all experiments. This may emphasize the fact that epithelial toxicity was most likely induced by the preservative (BAC) than by the active antiallergic compound in the present study too.

These findings strongly support the use of preservative-free solutions in patients with chronic eye diseases and treatments over the long-term, especially in allergic conjunctivitis or dry eye conditions. Definitely, preservative-free antiallergic medications may decrease the adverse effects of chronic topical medications, which could lead to better tolerability, lower treatment discontinuations and improved quality of life of patients with ocular allergic diseases.
